# Cost-Effectiveness and Budget Impact Analysis of the Trivalent Adjuvanted Influenza Vaccine in People over 50 Years of Age for Argentina

**DOI:** 10.3390/vaccines14030227

**Published:** 2026-02-28

**Authors:** Leandro Javier Pastori, Constanza Silvestrini Viola, Tomas Alconada, Gonzalo Pereira, José Luis Montes, Joaquin Mould-Quevedo, Carolina Saenz, Nathalia Katz, Ariel Bardach, Natalia Espinola

**Affiliations:** 1 Department of Health Technology Assessment and Health Economics, Institute for Clinical Effectiveness and Health Policy (IECS), Ciudad Autónoma de Buenos Aires C1414CPV, Argentinanespinola@iecs.org.ar (N.E.); 2 Commercial Operations, CSL Seqirus, Olivos C1636, Argentina; 3 Medical Affairs Latin America, CSL Seqirus, Olivos C1636, Argentina; 4 Global Medical Affairs, CSL Seqirus USA, Summit, NJ 07901, USA; 5 Center for the Study of the Prevention and Control of Communicable Diseases, ISALUD University, Ciudad Autónoma de Buenos Aires C1429, Argentina; nathaliakatz@gmail.com; 6 Centro de Investigaciones Epidemiológicas y Salud Pública (CIESP-IECS), CONICET, Ciudad Autónoma de Buenos Aires C1095AAS, Argentina

**Keywords:** adjuvanted influenza vaccine, influenza, economic evaluation, health care costs, preventive vaccination strategy, middle-income country, cost-effectiveness

## Abstract

Background: Influenza imposes a substantial burden on Argentina, particularly among adults aged 50–64 with comorbidities and those aged ≥65. The adjuvanted trivalent influenza vaccine (aTIV) has shown superior effectiveness compared with non-adjuvanted vaccines; however, its cost-effectiveness and budget impact in the 50–64 high-risk population have not been assessed nationally. This study evaluates the cost-effectiveness and budget impact of introducing aTIV for high-risk adults aged 50–64, alongside its use in adults aged ≥65, compared with standard-dose trivalent influenza vaccine (SD-TIV) from the Argentine health care system perspective. Methods: A decision-analytic static model was used to compare aTIV with SD-TIV over a single influenza season. In addition, a 5-year budget impact analysis (BIA) was conducted under scenarios of progressive uptake. Model inputs were derived from international literature, local data, and expert opinion. Deterministic and probabilistic sensitivity analyses were performed. Results: Compared with SD-TIV, aTIV yielded a lifetime gain of 1489 quality-adjusted life-years (QALYs) at an incremental cost of USD 8.34 million, resulting in an incremental cost-effectiveness ratio (ICER) of USD 5599 per QALY gained—well below Argentina’s cost-effectiveness threshold (USD 11,059/QALY). Higher vaccine acquisition costs were largely offset by reductions in outpatient visits and hospitalizations. The BIA showed a modest average annual per-member-per-month increase of USD 0.0025, remaining below the estimated budget impact threshold (USD 0.0065). Conclusions: Implementing aTIV in adults aged ≥50 with risk factors would be cost-effective and affordable in Argentina. These findings support the consolidation and potential expansion of current vaccination strategies to reduce influenza burden.

## 1. Introduction

Seasonal influenza is an acute respiratory infection and represents the leading cause of seasonal morbidity and mortality worldwide [1]. It is estimated to affect up to one billion people each year, resulting in 3 to 5 million severe cases and between 290,000 and 650,000 respiratory deaths attributable to the disease [1,2]. The greatest burden falls on adults aged 65 years and older, who account for nearly 90% of deaths, making it a major global public health concern [3].

Influenza epidemics in Argentina occur mainly during the winter and are caused by influenza A and B viruses, which circulate annually in different subtypes and lineages. Two predominant subtypes of influenza A (H1N1 and H3N2) circulate annually, while influenza B viruses consist of two lineages, Victoria and Yamagata [4,5]. Although most individuals recover within approximately one week without requiring medical care, certain high-risk groups may experience severe complications or even death. These groups include pregnant women, children under 5 years of age, older adults, individuals with chronic conditions (such as HIV/AIDS, asthma, heart disease, chronic lung disease, diabetes, obesity and smokers, among others), and those with greater occupational exposure to the virus, particularly healthcare workers [1,2].

A multicenter study conducted in six South American countries estimated that, between 2015 and 2019, influenza caused 51 to 78 million mild to moderate cases annually, along with 323,000 to 490,000 hospitalizations and 23,000 to 47,000 deaths [6,7]. Similarly, a systematic review covering several Latin American and Caribbean countries—including Argentina, Brazil, Chile, Cuba, and Mexico—reported an annual incidence of influenza-like illness (ILI) of 36,080 episodes per 100,000 population, with the highest burden observed in children under five years of age (45,730 per 100,000) [8]. In Argentina specifically, the annual burden is estimated at 3500 cases of ILI per 100,000 population, with 15.5 hospitalizations and 0.32 deaths per 100,000 adults aged 65 years and older [8,9].

Influenza also generates a substantial economic burden, involving both direct and indirect costs. In the United States, Putri et al. estimated that in 2015 the annual economic cost reached USD 11.2 billion, with indirect costs (USD 8 billion) outweighing direct costs (USD 3.2 billion) [10,11]. Complementarily, a recent systematic review in adults aged 18 to 64 years reported that up to 88% of the economic burden was attributable to indirect costs, while within direct costs, as much as 75% was associated with hospitalizations [10,11].

Preventive vaccination is a key strategy to reduce both the health and economic burden associated with influenza. The Centers for Disease Control and Prevention estimated that during the 2019–2020 season in the United States, influenza vaccination prevented 3.7 million outpatient visits, more than 100,000 hospitalizations, and approximately 6000 deaths [7]. Similarly, a study in the pediatric population in India estimated that the cost of vaccination was 7.76 times lower than the cost of treatment [11]. Currently, 41 countries and territories in the Americas provide influenza vaccination to high-risk groups defined at the national level [12]. In Argentina, influenza vaccination has been included in the National Immunization Schedule since 2011 for high-risk groups, including children 6 months to 2 years old, adults aged 65 years and older, healthcare workers, pregnant women, postpartum women not vaccinated during pregnancy, and individuals aged 2 to 64 years with comorbidities or chronic conditions, such as chronic respiratory disease, cardiovascular disease, diabetes, morbid obesity and immunosuppression, among others [13,14,15]. In Argentina, several influenza vaccines are approved by the National Administration of Drugs, Food and Medical Technology (ANMAT, Acronym in Spanish), including the standard-dose trivalent vaccine (SD-TIV), the standard-dose quadrivalent vaccine, cell culture trivalent vaccine, the high-dose quadrivalent vaccine (HD-QIV), and the adjuvanted trivalent vaccine [16]. However, no global circulation of the B/Yamagata lineage has been detected since 2020, and the World Health Organization currently recommends the use of trivalent influenza vaccines [17,18]. Consistent with these observations, data from official national reports indicate that in Argentina, only influenza B Victoria lineage viruses were detected during 2022, with no influenza B Yamagata lineage viruses identified since late 2019 [19].

Particularly, the aTIV vaccine combines the MF59^®^ adjuvant (an oil-in-water emulsion based on squalene) with a standard antigen dose, providing a stronger and longer-lasting immune response than standard-dose vaccines without adjuvant [3,20,21]. Multiple studies have shown that enhanced vaccines (either adjuvanted or high-dose) are more effective than standard vaccines in older adults [1,22,23]. Beyond clinical effectiveness, economic evaluations such as cost-effectiveness and budget impact analyses are widely recognized as key tools to support decision-making on the introduction of new health technologies [24]. In addition, aTIV has consistently been shown to be a cost-effective strategy in this population. In Canada, its use in individuals over 65 years was found to be highly cost-effective compared with SD-TIV [25]. Similarly, in Italy, aTIV showed the most favorable economic profile among the strategies evaluated, proving highly cost-effective both compared with no vaccination and with the SD-TIV [26]. Recently, in Spain, a study conducted in adults over 50 years of age at high risk of influenza complications evaluated the cost-effectiveness of vaccination with aQIV compared to HD-QIV. The results indicated that aQIV was a cost-saving alternative, and since the Yamagata lineage is no longer circulating, aQIV can be considered equivalent to aTIV [27]. Evidence from Argentina also supports the use of aTIV in older adults. One study showed that introducing aTIV for adults over 65 would be highly cost-effective compared to SD-TIV, with an incremental cost of USD 2660 per QALY gained [9]. Another study demonstrated that vaccination with aTIV in this population would be a dominant strategy over the HD-QIV [13].

Traditionally, aTIV has been recommended for individuals aged 65 and older. However, growing evidence suggests that extending influenza vaccination recommendations to include adults aged 50–64 years could broaden population coverage and help reduce the economic and social burden of influenza [22,28,29,30]. Reflecting this, the indication for aTIV has been expanded in recent years. In the United Kingdom, starting from the 2025–2026 influenza season, the Joint Committee on Vaccination and Immunisation (JCVI) has approved the use of aTIV for adults aged 50 to 64 in at-risk groups, as part of the national influenza vaccination program [31]. In line with these international developments, aTIV was recently approved in Argentina as an enhanced influenza vaccine for individuals aged 50–64 years, a group with a substantial disease burden that could potentially benefit from this strategy.

In this context, adults aged 50–64 years with risk factors experience substantial morbidity and healthcare utilization due to influenza. According to a report from the Argentine Ministry of Health, individuals aged 50–64 years had a three-times higher rate of hospitalization and a nine-fold higher mortality rate attributable to influenza than those aged 18–49-years, generating higher influenza-related hospitalization costs and highlighting the importance of reinforcing and optimizing preventive strategies within this population [32].

Although previous studies in Argentina have demonstrated the cost-effectiveness of aTIV compared with other influenza vaccines, the evidence is limited to adults aged ≥65 years. Consequently, the health and economic impact of using enhanced vaccines in adults aged 50–64 years with risk factors remains uncertain. To address this evidence gap and support the development of evidence-based vaccination policies, this study evaluates the cost-effectiveness and budget impact of implementing the aTIV influenza vaccine in people aged ≥50 years with risk factors, from the perspective of the Argentine healthcare system.

## 2. Materials and Methods

### 2.1. Model Structure

**Figure 1:**
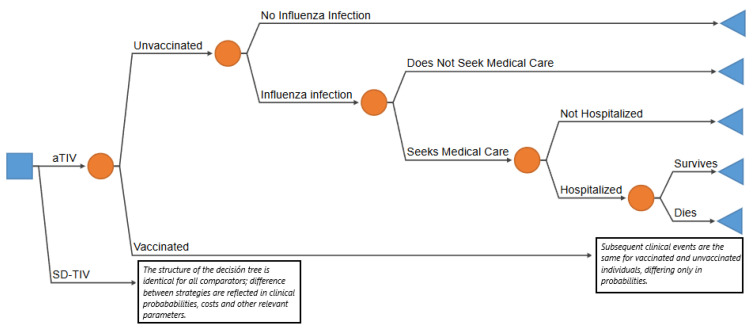
Analytical model.

A decision tree model, based on previously published economic evaluations, was adapted to compare the cost-effectiveness of aTIV with SD-TIV over a single influenza season in adults aged ≥50 years in Argentina (Figure 1) [27]. Individuals could either receive or not receive vaccination, after which both groups faced different risks of experiencing an influenza infection. Among those infected, individuals could either seek medical care or not; those who sought care could require hospitalization, and hospitalized patients could either survive or die. The structure of the decision tree was identical for both strategies, differing only in clinical event probabilities and cost parameters. Influenza-related outcomes include the number of symptomatic influenza cases, outpatient visits, hospitalizations, and deaths. Expected costs and health outcomes—life years (YLs) and quality-adjusted life-years (QALYs)—were estimated for each alternative to derive the incremental cost-effectiveness ratio (ICER). The time horizon for all model outcomes was a single influenza season (one year), except for YLs and QALYs, which were estimated over a lifetime horizon. A 5% annual discount rate was applied to YLs and QALYs only, in line with local guidelines [33,34].

In addition, a budget impact model was developed to estimate the 5-year financial impact of introducing aTIV for individuals aged ≥50 years within the healthcare system. The development of the budget impact analysis adhered to the principles of good practice in budget impact modeling, as outlined by the International Society for Pharmacoeconomics and Outcomes Research (ISPOR) task force [35].

The analysis was conducted for the Argentine population aged 50 years and older, considering adults aged 50–64 years with high risk to influenza (representing 36% of this age group), and adults aged 65 years and older [36,37]. The proportion of high-risk individuals aged 50–64 years was estimated taking into account influenza risk factors, including class III obesity, heart disease, myocardial infarction, COPD, cancer, leukemia, diabetes, kidney disease, and kidney transplant. The population is divided into four age groups: 50–59 years, 60–64 years, 65–74 years, and over 75 years.

Both models were estimated from the healthcare system. In Argentina, the healthcare system is fragmented into three sectors: public, social security, and private [38]. In the Argentine population over 50 years old (target population), health coverage is distributed as follows: 63% are covered by the public sector, 23% by social security, and 14% by the private sector [39,40]. Furthermore, 68% of the population covered by the public sector is part of the National Institute of Social Services for Retirees and Pensioners (PAMI, acronym in Spanish), which is the largest provider of health care for retirees and pensioners in the country.

The SD-TIV is available across all healthcare sectors; therefore, in the base-case analysis, aTIV was considered the intervention and SD-TIV the comparator, adopting the perspective of the overall Argentine healthcare system. An alternative scenario was evaluated, in which aTIV was used as the intervention for individuals aged ≥50 years and the comparator consisted of TIV-SD for those aged 50–64 years and HD-QIV for those aged ≥65 years. HD-QIV is currently being phased into the country’s private sector and is currently approved only for individuals aged ≥65 years. Therefore, this alternative scenario was included to capture a combined strategy that might be feasible exclusively in the private sector.

### 2.2. Model Parameters

The parameters required for the model were collected from various national public sources. In addition, high-quality international evidence validated as applicable to the Argentine context was used, and for certain parameters, local expert opinion was incorporated. A description of these parameters and their sources is presented below, while a detailed list of all parameters is provided in the Supplementary Material (SM).

#### 2.2.1. Epidemiological and Clinical Inputs

In Table 1 the main epidemiological and clinical parameters considered in the model are presented. The proportion of high-risk individuals aged 50–64 years, as well as vaccine coverage in this group and in those aged ≥65 years, were obtained from primary data of the second National Nutrition and Health Survey (ENNyS 2), conducted in 2018–2019 [37]. High-risk individuals were modeled as a single aggregated risk group, without differential weighting across specific conditions. Influenza attack rates were set at 11% for individuals aged 50–64 years and 6.3% for those aged ≥65 years, based on estimates reported by Urueña (2024), which were derived from ILI cases and viral isolates reported to the Argentine national surveillance system by age group [13]. The months of influenza circulation and the distribution of total cases by influenza A and B were derived from World Health Organization’s FluNet and the Directorate of Health Statistics and Information (DEIS) of the Argentine Ministry of Health, using the average from the 2023 and 2024 seasons [4,41]. The probabilities of outpatient visits and hospitalizations were derived from international literature, while the influenza case fatality ratio was obtained from hospital reports [41,42,43,44,45]. More detailed information is provided in Supplementary Materials Table S1.

The model incorporates vaccine efficacy for preventing influenza infection (Supplementary Materials Table S2). For influenza A (H1N1 and H3N2), age-stratified absolute efficacy estimates were used: 50–64 years and ≥65 years [46]. For influenza B, absolute efficacy in adults aged 50–64 years was obtained from Tricco et al. (2013), while absolute efficacy in adults ≥65 years was estimated by applying the relative efficacy ratio between younger and older adults reported by MacRondy et al. [47,48]. The efficacy of HD-QIV was calculated based on the relative efficacy of TIV-HD versus TIV-SD in preventing influenza cases among individuals aged ≥65 years, as reported by Diaz Granados et al. [49]. The absolute efficacy of aTIV was then derived from the estimated absolute efficacy of HD-QIV, using the relative efficacy reported by Hsiao et al. in a recent pragmatic trial, which found no difference between the two enhanced vaccines, consistent with findings from other studies [23,50,51,52]. For the 50–64 years age group, and in line with a previous economic evaluation published in 2025 by Perez-Rubio et al., the relative efficacy of aTIV was assumed to be equal to that observed in individuals aged ≥65 years, as no age-specific effectiveness estimates are currently available for the former age group [27].

#### 2.2.2. Utilities

Age-specific utility values for the Argentine population were obtained from Szende et al. (2014) [53]. The disutilities and durations for symptomatic influenza cases who did not seek care, outpatient visits, and hospitalizations were collected from Bilcke et al. (2014) and Hollman et al. (2013) [54,55]. See Supplementary Materials Table S3.

#### 2.2.3. Costs

The cost categories included in both models were vaccine acquisition and administration costs, outpatient visit costs, and hospitalization costs.

The acquisition costs of aTIV and HD-QIV for the social security sector and the private sector were obtained from public databases that provide the retail prices of drugs and converted to wholesale prices using a conversion factor obtained from the Ministry of Economy report (ex-factory price) [56,57]. There are two SD-TIV brands marketed in Argentina; however, during the influenza season analyzed, only the Seqirus brand (AGRIPPAL^®^ S1, SEQIRUS CSL, Buenos Aires, Argentina) was available in both the social security and private healthcare sectors, and it did not have published prices. In this context, the ex-factory price was provided by Seqirus. For the public sector, the vaccine acquisition cost for the SD-TIV and aTIV was obtained from data of tender price of National Procurement Office of Argentina [58]. The prices of all vaccines are presented in Table 2.

We used a micro-costing approach to estimate the remaining cost categories. The unit cost of healthcare resources by sector was obtained from the IECS unit cost database; drug acquisition costs were calculated using ex-factory prices and assumed to be the same across the three perspectives of analysis, and the expected quantities of each resource were obtained through local expert opinion [56,57,59]. For more information, see Supplementary Materials (Tables S4–S6).

To obtain the direct medical costs for the health system, an average weighted by coverage rates of each sector was used. All costs were estimated in Argentinian pesos (ARS) and then converted to US dollars (US$) for March 2025 (1 US$ = $1066.50) [60].

**Table 2:** Cost of vaccine acquisition per dose in Argentina. Costs are reported in March 2025 USD dollars.

Vaccine	Public/PAMI Sector *	Social Security	Private Sector	Health System ^††^
Vaccine acquisition				
SD-TIV	$2.38	$26.72 **	$26.72 **	$11.39
aTIV	$6.41	$29.05 ***	$29.05 ***	$14.78
HD-QIV	-	-	$34.09	-
Vaccines Administration ^†^	$1.32	$2.68	$3.49	$1.93
Outpatient Visit	$28.58	$43.97	$46.98	$34.70
Hospitalization	$2599.60	$6006.51	$6566.46	$3938.55

Abbreviations. SD-TIV, standard-dose trivalent. aTIV, adjuvanted trivalent. HD-QIV, high-dose quadrivalent. * Final price without Value Added Tax (IVA, its acronym in Spanish) obtained from the 2024 public tenders of the Ministry of Health (MSAL) COMPRAR [58]. ** Price provided by Seqirus. *** Retail price (PVP) obtained from ALFABETA and converted into ex-factory price (PSL) by dividing the retail price by 1.7545 [56,57]. ^†^ The administration cost was calculated based on the wage corresponding to 10 min of a nurse’s work [61]. ^††^ To estimate the direct medical costs for the health system, a weighted average was used based on the coverage rates of each sector (23% social security, 14% private sector, and 63% public sector).

#### 2.2.4. Market Share Rates

For the budget impact model, current and projected market shares over a 5-year time horizon were estimated for the different vaccination regimens, and age groups (50–64 years with high risk to influenza and ≥65 years) based on estimates provided by Seqirus and validated by a local expert, reflecting the reality of each health subsector. Market share data are presented in Supplementary Materials Tables S7 and S8.

### 2.3. Decision Rule

In Argentina, there are currently no explicit decision rules to evaluate the cost-effectiveness and budget impact of incorporating new health technologies. Therefore, in our analysis we adopted the reference cost-effectiveness threshold (CET) proposed by Pichon-Riviere et al. and updated to March 2025 [62]. This CET is estimated using macroeconomic indicators—gross domestic product, health expenditure, and population size—resulting in $11,059 per QALY for 2025.

In the case of budget impact analysis, this study employed the methodology utilized by the National Commission for Health Technology Assessment and Clinical Excellence of the Ministry of Health (CONETEC, acronym in Spanish), the local Health Technology Assessment, in the country, to estimate a threshold of high budgetary impact. This approach is grounded in the study of Pichón-Riviere et al., which is particularly relevant for countries lacking their own estimates [63,64]. Four categories of budget impact thresholds were defined as the three budgetary impact thresholds as a proportion of per capita health expenditure: low (<0.00005), moderate (0.00005 to <0.0001), high (0.0001 to <0.0002), and very high (≥0.0002). Using these reference values and updating the estimation of per capita health expenditure to March 2025, using data from World Bank and national data [65], the budget impact thresholds for the Argentine health system were estimated and expressed in terms of per member per month (PMPM) incremental cost, representing the average monthly incremental cost per covered individual. As a result, the monthly per-member budget impact (PMPM) thresholds for the Argentine health system were estimated as follows: low < $0.0063; moderate [$0.0063–$0.0125); high [$0.0125–$0.0250); and very high ≥ $0.0250. The results are presented as absolute incremental costs, incremental costs as a percentage of current budget, and incremental costs PMPM. Both threshold values should be regarded as a reference value.

### 2.4. Sensitivity Analysis

Multiple one-way deterministic sensitivity analyses (DSA) were conducted using a tornado diagram to evaluate the impact of parameter uncertainty on the ICER. When confidence intervals were not available, a ±20% variation around the base-case value was applied to clinical and cost parameters. In addition, a probabilistic sensitivity analysis (PSA) was conducted, varying clinical input parameters according to their confidence intervals across 10,000 iterations. The variables included and their distributions are detailed in Supplementary Materials Table S9.

## 3. Results

### 3.1. Cost-Effectiveness Analysis

In the base-case scenario for the Argentine population, it is estimated that aTIV prevented 38,111 symptomatic influenza cases, 16,485 outpatient visits, 1182 hospitalizations, and 176 deaths in individuals aged ≥50 years (50–64 years at high risk for influenza and ≥65 years), compared to SD-TIV. This strategy would also generate savings of $572,000 in outpatient visits and $4 million in hospitalizations, while incurring an incremental vaccination cost of approximately $13 million (Table 3). See Supplementary Material Table S10 for the results of the alternative scenario.

Compared with SD-TIV, the aTIV vaccination strategy resulted in a reduction in QALYs lost due to illness by 1488.88 (15,777.77 vs. 17,266.65), and an incremental cost of $8,335,646 ($121,940,020 vs. $113,604,374) (Table 4). This corresponds to an ICER of $5599 per QALY gained, making it a cost-effective strategy at a CET of $11,059 per QALY.

In the alternative scenario for the private healthcare sector, aTIV showed a gain of 57.93 QALYs and cost savings of $1,211,567, resulting in a dominant strategy, compared with the combined strategy SD-TIV for adults 50–64 years and HD-QIV for adults ≥65 years (See in Supplementary Materials, Table S11).

#### Sensitivity Analysis

**Figure 2:**
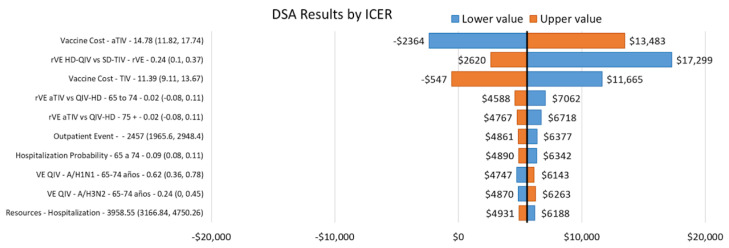
Tornado diagram for the base-case analysis in the population aged 50–64 years at high risk for influenza and ≥65 years.

**Figure 3:**
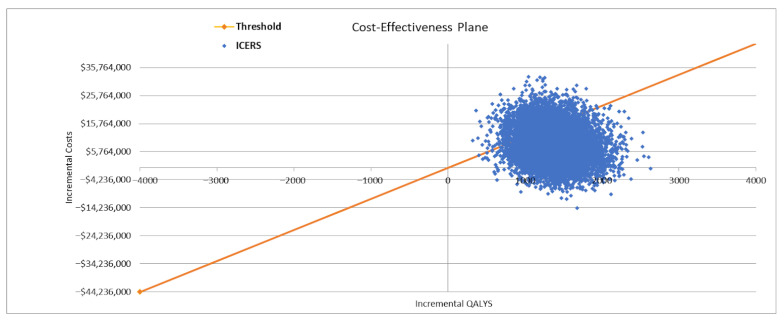
Cost-effectiveness plane for the base-case analysis in the population aged ≥50 years *: aTIV vs. SD-TIV. * 50–64 years at high risk for influenza and ≥65 years.

**Figure 4:**
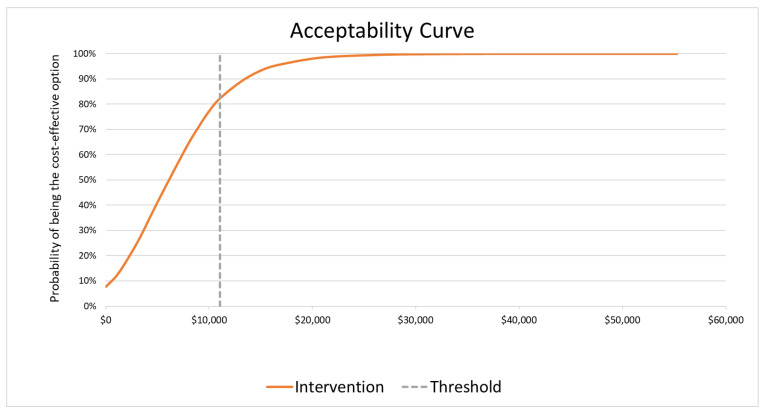
Cost-effectiveness acceptability curve for the base-case analysis in the population aged ≥50 years *: aTIV vs. SD-TIV. * 50–64 years at high risk for influenza and ≥65 years.

The results of DSA for the base-case scenario are presented in Figure 2. The cost of the aTIV vaccine had the greatest impact on the ICER, followed by the relative effectiveness of HD-QIV versus QIV and the cost of SD-TIV. The PSA for the base-case scenario showed that the results are robust, with the intervention being cost-effective in 78% of simulations at a reference CET (Figure 3 and Figure 4).

The DSA and PSA for the alternative scenario and the cost-effectiveness acceptability curves for both the base-case and scenario 1 are provided in the Supplementary Material Figures S1–S4.

### 3.2. Budget Impact Analysis

**Figure 5:**
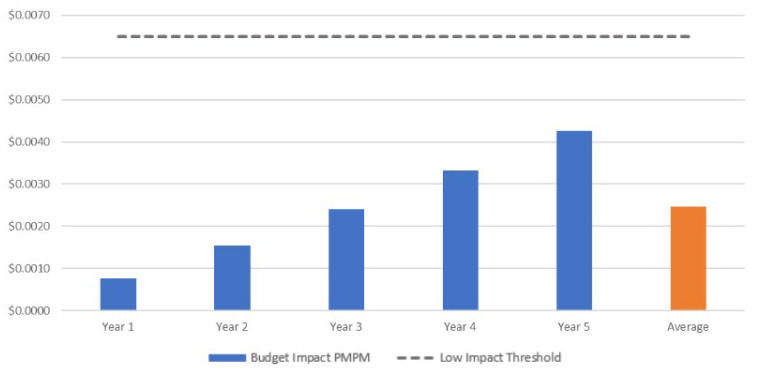
Budget impact per-member per-month (PMPM) for the base-case analysis in the population aged ≥50 years *. * 50–64 years at high risk for influenza and ≥65 years.

Table 5 presents the results of the budget impact analysis for the base-case analysis in the population aged ≥50 years (50–64 years at high risk for influenza and ≥65 years), disaggregated by cost component and year. The average annual incremental cost was $1,400,135, representing 1.20% of the current budget scenario. The increase in costs is primarily attributable to the higher acquisition cost of the vaccines, although savings were observed in outpatient visits and hospitalizations. On average, the introduction of aTIV in the 50–64 years group and its expansion in the ≥65 years group increased costs by $0.0025 per member per month (PMPM) for the national health system, based on the market share distribution assumed in the analysis. In other words, this means that an additional $0.0025 would need to be allocated per affiliate in the health system to cover the new technology. This incremental cost per member remains below the estimated low national budget impact threshold (<$0.0063) (Figure 5).

In the scenario analysis for the private healthcare sector, the average annual incremental budget impact was $13,113, representing a 0.05% increase over the current budget, according to the market share scenario applied. On average, the introduction of aTIV increased costs by $0.00015 per member per month for the private sector, remaining below the estimated low national budget impact threshold (<$0.0063) (Supplementary Materials Table S10).

## 4. Discussion

Our analysis demonstrates that implementing aTIV in adults aged 50–64 with risk factors, as well as in adults over 65, is a cost-effective strategy in Argentina. Compared with SD-TIV, aTIV would result in an incremental cost of $8,335,646 and a gain of 1489 QALYs, yielding an ICER of $5599 per QALY, well below the estimated cost-effectiveness threshold for the country ($11,059/QALY). The higher vaccine acquisition cost is offset by reductions in outpatient visits and hospitalizations. The BIA showed that, over a 5-year horizon, progressively introducing aTIV in individuals aged 50–64 years with risk factors for influenza up to 40% coverage, together with expanding coverage from 55% to 80% in those aged ≥65 years, would lead to an average annual PMPM increase of $0.0025 for the national health system, remaining within the range corresponding to a low national budget impact (<$0.0063). In addition to being a potentially cost-effective strategy, these results indicate that implementing aTIV would have a low impact on the national health system budget. The interpretation of this budget impact threshold refers to the health system as a whole; in fragmented health systems such as Argentina’s, it should be adapted to the relevant subsector or payer [64].

These findings are consistent with prior evidence from multiple countries and Argentina, which has consistently demonstrated the cost-effectiveness of aTIV in older adults. While most previous studies focused on adults aged ≥65, our results indicate that extending the use of aTIV to high-risk adults aged 50–64 could further reduce the health and economic burden of influenza. This alignment with international and local evidence reinforces the potential value of aTIV as a preventive strategy across a broader high-risk population.

This study has certain limitations that should be acknowledged. The most important is the current lack of data on vaccine efficacy and effectiveness in adults aged 50–64 years, which requires extrapolation from populations aged ≥65 years and introduces additional uncertainty in the effectiveness parameter used in the model. In addition, a static model was selected to ensure parsimony and comparability with previous regional economic evaluations; however, as this approach does not account for indirect effects, the estimated health benefits and cost-effectiveness of expanding vaccination coverage, particularly in younger age groups, are likely to be conservative.

Another limitation is the lack of precise national, age-specific estimates of influenza incidence. Although Urueña et al. used reports from the national public surveillance system, these data were carefully adjusted for healthcare subsector coverage, viral isolation, vaccination coverage, and vaccine effectiveness, which may introduce uncertainty.

A further limitation is the lack of local data on health-seeking behavior in response to influenza-like illness. This parameter is highly context-dependent and closely linked to the idiosyncrasies of the population, so relying on international evidence may not fully reflect local patterns. Nevertheless, we relied on a meta-analysis that included data from several countries, focusing exclusively on observations made after the 2009 pandemic, which makes it a solid and appropriate choice for this parameter. Another limitation is the absence of interannual variability in the model, including potential pandemic peaks or changes in viral circulation, which could influence the epidemiological and economic outcomes. Finally, indirect costs related to productivity losses were not included; in populations aged 50–64 years, these costs may be substantial, suggesting that the economic benefits of vaccination could be underestimated.

From a health policy perspective, our findings suggest that introducing aTIV in adults aged 50 and older with prevalent comorbidities—such as COPD, diabetes, or immunocompromising conditions—could be a cost-effective strategy, substantially reducing influenza-related morbidity and mortality while easing pressure on hospital services during periods of high viral circulation. From a public health perspective, expanding coverage with aTIV could improve the efficiency of the National Immunization Program, in line with international recommendations, which advise aTIV for adults aged 65 years and older, with some countries now beginning to extend this recommendation to those aged 50–64 [66,67,68,69,70]. For payers, the analysis indicates that the additional cost is relatively modest in relation to the overall health budget and is partially offset by savings in hospitalizations and outpatient visits. In a resource-constrained setting, prioritizing technologies that combine economic efficiency with a meaningful impact on population health is critical for ensuring the sustainability of the healthcare system.

Despite the robustness of this analysis, some questions remain that could be addressed in future research, such as evidence on the effectiveness of aTIV in adults aged 50–64 years and age-specific health-seeking behavior. It would also be valuable to incorporate indirect costs related to productivity losses into future models, given the relevance of this aspect for the 50–64 age group. Finally, assessing the distributive impact of introducing aTIV within a fragmented health system will be essential to identify potential inequities in access. Advancing research in these areas will help strengthen the evidence base and inform policy decisions that maximize the clinical and economic benefits of influenza vaccination in Argentina.

## 5. Conclusions

In conclusion, this study provides the first economic evaluation of aTIV in Argentina for adults aged 50 and older, showing that its introduction would be cost-effective while maintaining a low impact on the budget of the payers in the health system, compared with SD-TIV. Expanding aTIV vaccination could reduce influenza-related morbidity, complications, and mortality, supporting its role as a strategic intervention to strengthen prevention policies from age 50 and improve population health outcomes.

## Figures and Tables

**Table 1:** Main epidemiological and clinical parameters.

Parameter	Base Case	Reference
Age cohort		
50–59 years	4,715,493	INDEC, 2024 [36]
60–64 years	1,994,318	INDEC, 2024 [36]
65–74 years	3,225,327	INDEC, 2024 [36]
+75 years	2,512,491	INDEC, 2024 [36]
High-risk individuals *		
50–64 years	36.04% (33.54–38.54%)	ENNyS 2, 2019 [37]
+65 years	100%	Assumption
Vaccination Coverage Rate		
50–64 years high-risk individuals	39.53% (36.41–44.45%)	ENNyS 2, 2019 [37]
65–74 years	51.65% (47.96–55.34%)	ENNyS 2, 2019 [37]
+75 years	58.37% (53.43–63.31%)	ENNyS 2, 2019 [37]
Strain Distribution		
Influenza B	10.83%	FluNet [4]
Influenza A H1N1	46.77%	FluNet [4]
Influenza A H3N2	42.40%	FluNet [4]
Influenza attack rates		
50–64 years	11% (8.25–13.75%	Urueña, 2024 [13]
+65 years	6.3% (4.75–7.90%)	Urueña, 2024 [13]

* Proportion estimated using the variables obesity III, heart disease, myocardial infarction, COPD, cancer, leukemia, diabetes, kidney disease, and kidney transplant from ENNyS 2.

**Table 3:** Base-case results for the Argentine population aged ≥50 years *.

Influenza Outcomes	aTIV	SD-TIV	Difference
**Clinical outcomes**			
Symptomatic cases	435,200	473,310	−38,111
Outpatient visits	197,454	213,940	−16,485
Hospitalizations	12,281	13,463	−1182
Deaths	1693	1868	−176
QALYs lost	15,777.77	17,266.65	−1488.88
**Economic outcomes**			
Vaccine acquisition	$59,000,956	$45,437,058	$13,563,899
Vaccines administration	$7,718,826	$7,718,826	$0
Outpatient visits	$6,851,242	$7,423,248	−$572,002
Hospitalizations	$48,368,995	$53,025,246	−$4,656,251

* 50–64 years at high risk for influenza and ≥65 years.

**Table 4:** Base-case results of cost-effectiveness analysis for the Argentine population aged ≥50 years.

	Total Costs	QALYs Lost	Incremental Costs	Incremental QALYs	ICER per QALY
aTIV	$121,940,020	15,777.77	$8,335,646	1488.88	$5599
SD-TIV	$113,604,374	17,266.65			

**Table 5:** Base-case scenario results in the population aged ≥50 years * presented for the current scenario, the projected scenario, and the corresponding budget impact. Costs are reported as March 2025 USD dollars.

Cost Component	Year 1	Year 2	Year 3	Year 4	Year 5	Cumulative	Annual Average
Current scenario							
Vaccine acquisition	$51,078,228	$51,078,228	$51,078,228	$51,078,228	$51,078,228	$255,391,139	$51,078,228
Vaccines administration	$7,718,826	$7,718,826	$7,718,826	$7,718,826	$7,718,826	$38,594,131	$7,718,826
Outpatient visits	$7,225,745	$7,225,745	$7,225,745	$7,225,745	$7,225,745	$36,128,725	$7,225,745
Hospitalizations	$50,917,949	$50,917,949	$50,917,949	$50,917,949	$50,917,949	$254,589,745	$50,917,949
Total cost	$116,940,748	$116,940,748	$116,940,748	$116,940,748	$116,940,748	$584,703,741	$116,940,748
Projected scenario							
Vaccine acquisition	$51,789,495	$52,500,762	$53,278,174	$54,121,730	$54,965,286	$266,655,448	$53,331,090
Vaccines administration	$7,718,826	$7,718,826	$7,718,826	$7,718,826	$7,718,826	$38,594,131	$7,718,826
Outpatient visits	$7,195,016	$7,164,287	$7,129,299	$7,090,053	$7,050,806	$35,629,463	$7,125,893
Hospitalizations	$50,676,888	$50,435,828	$50,178,271	$49,997,983	$49,630,166	$250,825,372	$50,165,074
Total cost	$117,380,226	$117,819,703	$118,304,571	$118,834,828	$119,365,086	$591,704,414	$118,340,883
Budget impact							
Vaccine acquisition	$711,267	$1,422,534	$2,199,946	$3,043,502	$3,887,059	$11,264,309	$2,252,862
Vaccines administration	$0	$0	$0	$0	$0	$0	$0
Outpatient visits	−$30,729	−$61,458	−$96,446	−$135,692	−$174,937	−$499,263	−$99,853
Hospitalizations	−$241,061	−$482,121	−$739,678	−$1,013,730	−$1,287,783	−$3,764,373	−$752,875
Incremental costs	$439,477	$878,955	$1,363,822	$1,894,080	$2,424,338	$7,000,673	$1,400,135
Incremental costs, as a % of current budget **	0.38%	0.75%	1.17%	1.62%	2.07%	5.99%	1.20%
PMPM ***	$0.0008	$0.0016	$0.0024	$0.0034	$0.0043	$0.0124	$0.0025

* 50–64 years at high risk for influenza and ≥65 years. ** Calculated as the projected scenario budget relative to the current scenario budget. *** Represents the average monthly incremental cost per covered individual. Abbreviations. PMPM, per-member per-month.

## Data Availability

The main manuscript and its supplementary information files include all data involved in this study.

## Supplementary Materials

The following supporting information can be downloaded at: https://www.mdpi.com/article/10.3390/vaccines14030227/s1, Table S1: Probabilities of Medical Visits And Hospitalizations; Table S2: Vaccine Efficacies; Table S3: Utilities Lost; Table S4 Cost of drugs included in the management of an outpatient and inpatient event; Table S5: Unit costs of healthcare resources used in the vaccine administration, in the management of an outpatient event and in the management of an inpatient event in Argentina; Table S6: Expected amounts per patient per year of each health and medication used in the vaccines administration, management of an outpatient and inpatient event; Table S7: Market share for the base-case scenario; Table S8: Market share for the scenario analysis; Table S9: Distribution of variables included in the probabilistic sensitivity analysis; Table S10: Discounted scenario analysis results; Figure S1: One-way sensitivity analysis; Figure S2: Probabilistic sensitivity analysis for the scenario analysis; Figure S3: Cost-effectiveness acceptability curve for the scenario analysis; Table S11: Budget impact for the scenario analysis; Figure S4: Budget impact per-member per-month (PMPM) for the scenario analysis.

## Abbreviations

The following abbreviations are used in this manuscript:

aTIV	Adjuvanted trivalent influenza vaccine
SD-TIV	Standard-dose trivalent influenza vaccine
QALYs	Quality-adjusted life-years
ICER	Incremental cost-effectiveness ratio
ILI	Influenza-like illness
HD-QIV	High-dose quadrivalent vaccine
YLs	Life years
PAMI	National Institute of Social Services for Retirees and Pensioners
ENNyS 2	Second National Nutrition and Health Survey
DSA	Deterministic sensitivity analyses
PSA	Probabilistic sensitivity analysis
PMPM	Per member per month
