# Supplementary material for: Cost-Effectiveness and Budget Impact Analysis of the Trivalent Adjuvanted Influenza Vaccine in People over 50 Years of Age for Argentina
Source: Vaccines (Basel). 2026 Feb 28;14(3):227. doi: 10.3390/vaccines14030227 (PMC13030660; doi:10.3390/vaccines14030227)
Supplement: Supplementary file 1 [file vaccines-14-00227-s001.zip › Supplementary Material.docx]

[**Supplementary Material 1**](#_v3hbzup9o6vx)

[**Epidemiological and clinical inputs 1**](#_4o15pl5g3uu)

[Table S1. Probabilities of Medical Visits and Hospitalizations 1](#_sa4rytb1s9bf)

[**Efficacies inputs 2**](#_g9x1o3ngibq0)

[Table S2. Vaccine Efficacies 2](#_3ynk4mqhgaha)

[**Utilities inputs 2**](#_dpiu5fek9jy8)

[Table S3. Utilities lost 2](#_czp001ye0f47)

[**Cost inputs 3**](#_fhz5xjy9cjju)

[Table S4. Cost of drugs included in the management of an outpatient and inpatient event. Costs are reported in March 2025 USD dollars. 3](#_naw3gt5ax5kg)

[Table S5. Unit costs of healthcare resources used in the vaccine administration, in the management of an outpatient event and in the management of an inpatient event in Argentina. Costs are reported in March 2025 USD dollars. 3](#_eezu0xkxh3gk)

[Table S6. Expected amounts per patient per year of each health and medication used in the vaccines administration, management of an outpatient and inpatient event. Expected quantities are reported according to phase of the health event. 4](#_xcv77o4xjgpo)

[**Market share for the base-case scenario 5**](#_rx3cs4bdvse7)

[Table S7. Market share for the base-case scenario. 5](#_l70p8r3u6emb)

[**Market share for the scenario analysis 6**](#_z38ghjjiwqvo)

[Table S8. Market share for the scenario analysis. 6](#_3n9o5oss4tn8)

[**Probabilistic Sensitivity Analysis (PSA) 7**](#_yfp29tfj586j)

[Table S9. Distribution of variables included in the probabilistic sensitivity analysis 7](#_glas79w2u0fq)

[**Results for the scenario analysis 7**](#_qejqwjjdg82j)

[Table S10. Discounted scenario analysis results. 7](#_goyjt3bciz1d)

[Figure 1. One-way sensitivity analysis for the scenario analysis. 8](#_ebp7gyq7l0ly)

[Figure 2. Probabilistic sensitivity analysis for the scenario analysis. 9](#_cml7m2dj7xb5)

[Figure 3. Cost-effectiveness acceptability curve for the scenario analysis. 9](#_bgko9r6nbxga)

[Table S11. Budget impact for the scenario analysis. Costs are reported in March 2025 USD dollars. 9](#_e1lzxaxkdw48)

[Figure 4. Budget impact per-member per-month (PMPM) for the scenario analysis. 10](#_6cog08ulk6xb)

# **Supplementary Material**

# **Epidemiological and clinical inputs**

## **Table S1. Probabilities of Medical Visits and Hospitalizations**

| **Parameter** | **Base Case** | **Lower bound** | **Upper bound** | **Distribution** | **Reference** |
| --- | --- | --- | --- | --- | --- |
| **Probability of medical visit*** | 39% | 33% | 45% |  | Ma W, 2018[^1^](https://paperpile.com/c/pda1Wq/mzUHE) & Fleming, 2016[^2^](https://paperpile.com/c/pda1Wq/E53cQ) |
| **Probability of hospitalization**** | | | | | |
| 50-64 years | 2.3% |  |  | Beta distribution | Near, 2022[^3^](https://paperpile.com/c/pda1Wq/rBvXU) & Mertz, 2013[^3,4^](https://paperpile.com/c/pda1Wq/rBvXU+3lH8I) |
| +65 years | 9.4% |  |  | Beta distribution |  |

*The probability of medical visit for a symptomatic influenza case was obtained from a meta-analysis conducted in 2018 and was adjusted using the study by Fleming et al. (2016) for the 50–64-year high-risk group for influenza.

**The probability of hospitalization among patients who sought care was obtained from a real-world study conducted in the United States and adjusted for the 50–64-year high-risk group for influenza.

# **Efficacies inputs**

## **Table S2. Vaccine Efficacies**

| **Parameter** | **Base Case** | **Lower bound** | **Upper bound** | **Distribution** | **Reference** |
| --- | --- | --- | --- | --- | --- |
| **50-64 years** |  |  |  |  |  |
| SD-TIV efficacy for H1N1 | 73% | 52% | 84% | Lognormal distribution | Belongia, 2016[^5^](https://paperpile.com/c/pda1Wq/ovlBJ) |
| SD-TIV efficacy for H3N2 | 35% | 14% | 51% | Lognormal distribution | Belongia, 2016[^5^](https://paperpile.com/c/pda1Wq/ovlBJ) |
| SD-TIV efficacy for B | 77% | 18% | 94% | Lognormal distribution | Tricco, 2013[^6^](https://paperpile.com/c/pda1Wq/cAgng) |
| QIV-HD vs TIV/QIV-SD for influenza cases | 24.2% | 9.7% | 36.5% | Lognormal distribution | Assumption |
| aTIV vs QIV-HD for influenza cases | 1.5% | -8.4% | 10.5% | Lognormal distribution | Assumption |
|  |  |  |  |  |  |
| **+65 years** |  |  |  |  |  |
| SD-TIV efficacy for H1N1 | 62% | 36% | 78% | Lognormal distribution | Belongia, 2016[^5^](https://paperpile.com/c/pda1Wq/ovlBJ) |
| SD-TIV efficacy for H3N2 | 24% | 0% | 45% | Lognormal distribution | Belongia, 2016[^5^](https://paperpile.com/c/pda1Wq/ovlBJ) |
| SD-TIV efficacy for B | 53% | 39% | 64.7% | Lognormal distribution | Tricco, 2013[^6^](https://paperpile.com/c/pda1Wq/cAgng) |
| QIV-HD vs TIV/QIV-SD for influenza cases | 24.2% | 9.7% | 36.5% | Lognormal distribution | DiazGranados, 2014[^7^](https://paperpile.com/c/pda1Wq/rwElR) |
| aTIV vs QIV-HD for influenza cases | 1.5% | -8.4% | 10.5% | Lognormal distribution | Hsiao, 2025[^8^](https://paperpile.com/c/pda1Wq/cYGxG) |

# **Utilities inputs**

## **Table S3. Utilities lost**

| **Parameter** | **Base Case** | **Lower bound** | **Upper bound** | **Distribution** | **Reference** |
| --- | --- | --- | --- | --- | --- |
| Symptomatic case | 0.30 | 0.00 | 0.53 | Beta distribution | Bilcke, 2014[^9^](https://paperpile.com/c/pda1Wq/7pjnt) |
|  |  |  |  |  |  |
| **50-64 years** |  |  |  |  |  |
| Medical visit | 0.36 | 0.29 | 0.43 | Beta distribution | Hollmann, 2013[^10^](https://paperpile.com/c/pda1Wq/0Irdn) |
| Hospitalization | 0.58 | 0.49 | 0.67 |  | Hollmann, 2013[^10^](https://paperpile.com/c/pda1Wq/0Irdn) |
|  |  |  |  |  |  |
| **+65 years** |  |  |  |  |  |
| Medical visit | 0.32 | 0.20 | 0.45 | Beta distribution | Hollmann, 2013[^10^](https://paperpile.com/c/pda1Wq/0Irdn) |
| Hospitalization | 0.56 | 0.43 | 0.69 |  | Hollmann, 2013[^10^](https://paperpile.com/c/pda1Wq/0Irdn) |

For the 50–69-year-old group, the utility was estimated as an average of the 45–54 and 55–64 age groups, weighted by the proportion of individuals aged 50–59 who were under 55, based on the 2024 population projections from INDEC.

# **Cost inputs**

## **Table S4.** **Cost of drugs included in the management of an outpatient and inpatient event.** Costs are reported in March 2025 USD dollars.

| **Health phases** | **Drugs** | **Dosage** | **Expected amount** | **Wholesale price per mg*** |
| --- | --- | --- | --- | --- |
| Management of an outpatient event | Acetaminophen | 1 gr every 12 hours | 8,400 mg | $0.0002 |
|  | Oseltamivir | 75 mg per day | 26,25 mg | $0.0305 |
| Management of an inpatient event | Piperacillin + tazobactam (IV) | 4.5gr every 6 hours | 25,200 mg | $0,0121 |
|  | Oseltamivir (O) | 75 mg per day | 197 mg | $0,0305 |
|  | Cefepime (IV) | 4.5 gr per day | 9,000 mg | $0,0248 |
|  | Vancomycin (IV) | 2 gr per day | 5,200 mg | $0,0289 |
|  | Sulfamethoxazole + trimethoprim (IV) | 1.6 vials of 80/400 mg per day | 64 mg | $0,0014 |
|  | Ampicillin sulbactam (IV) | 1.5 gr every 6 hours | 18,000 mg | $0,0076 |
|  | Ceftriaxone (IV) | 1.5 gr per day | 7,350 mg | $0,0117 |
|  | Levofloxacin (IV) | 500 mg every 12 hours | 1,225 mg | $0,0027 |
|  | Clarithromycin (IV) | 500 mg every 12 hours | 2,100 mg | $0,0022 |

*The retail price was obtained from ALFA BETA, and the ex-factory price was estimated as the retail price divided by 1.7545.[^11,12^](https://paperpile.com/c/pda1Wq/j6RVw+ILhgw) ** This number is obtained by multiplying the daily dose by the number of days and by the utilization rate.

**Abbreviations**. IV, Intravenous. O, Orally.

## **Table S5. Unit costs of healthcare resources used in the vaccine administration, in the management of an outpatient event and in the management of an inpatient event in Argentina.** Costs are reported in March 2025 USD dollars.

| **Health resource** | **Public/ PAMI sector** | **Social Security** | **Private sector** | **Health System** |
| --- | --- | --- | --- | --- |
| Consultation with a general practitioner | $9.72 | $11.56 | $12.42 | $10.52 |
| Consultation with pulmonologist | $9.72 | $11.56 | $12.42 | $10.52 |
| Consultation with an infectious disease specialist | $9.72 | $11.56 | $12.42 | $10.52 |
| Vaccine administration* | $0.09 | $0.18 | $0.23 | $0.13 |
| Blood culture | $10.11 | $24.83 | $26.25 | $15.76 |
| Sputum culture | $12.32 | $36.52 | $38.60 | $21.57 |
| Respiratory viral panel | $46.50 | $131.48 | $138.96 | $78.99 |
| Urea | $1.43 | $2.19 | $2.32 | $1.73 |
| Creatinine | $4.07 | $2.92 | $3.09 | $3.67 |
| Hepatogram | $3.71 | $8.77 | $9.26 | $5.65 |
| Blood glucose | $0.78 | $2.19 | $2.32 | $1.32 |
| Ion panel | $2.40 | $5.11 | $5.40 | $3.44 |
| Blood count | $1.62 | $4.38 | $4.63 | $2.68 |
| C-reactive protein | $2.34 | $17.53 | $18.53 | $8.10 |
| Bronchoalveolar lavage | $29.06 | $9.27 | $15.96 | $22.67 |
| Chest X-ray | $6.25 | $7.93 | $8.79 | $6.99 |
| Chest tomography | $49.04 | $53.80 | $64.60 | $52.31 |
| General ward admission | $100.56 | $325.40 | $358.05 | $188.32 |
| Intensive care unit admission | $169.43 | $548.27 | $618.86 | $319.49 |
| Mechanical Ventilation | $185.95 | $601.70 | $665.66 | $348.73 |

The unit cost of healthcare resources by sector was obtained from the IECS unit cost database.

*****The administration cost was calculated based on the wage corresponding to 10 minutes of a nurse’s work.[^13^](https://paperpile.com/c/pda1Wq/H7Klp)

**To estimate the direct medical costs for the health system, a weighted average was used based on the coverage rates of each sector (23% social security, 14% private sector, and 63% public sector).

## **Table S6. Expected amounts per patient per year of each health and medication used in the vaccines administration, management of an outpatient and inpatient event.** Expected quantities are reported according to phase of the health event.

| **Health phases** | **Health resource** | **Expected amounts*** |
| --- | --- | --- |
| Vaccines administration | Nurse | 10.00 |
| Management of an outpatient event | Consultation with a general practitioner | 1.50 |
|  | Urea | 0.40 |
|  | Creatinine | 0.40 |
|  | Hepatogram | 0.40 |
|  | Blood glucose | 0.40 |
|  | Ion panel | 0.40 |
|  | Blood count | 0.40 |
|  | C-reactive protein | 0.40 |
|  | Respiratory viral panel | 0.01 |
|  | Chest X-ray | 0.60 |
|  | Chest tomography | 0.015 |
| Management of an inpatient event | Consultation with pulmonologist | 2.00 |
|  | Consultation with an infectious disease specialist | 4.00 |
|  | Consultation with a general practitioner | 2.00 |
|  | Blood culture | 0.90 |
|  | Sputum culture | 0.40 |
|  | Respiratory viral panel | 0.35 |
|  | Urea | 2.00 |
|  | Creatinine | 2.00 |
|  | Hepatogram | 2.00 |
|  | Blood glucose | 2.00 |
|  | Ion panel | 2.00 |
|  | Blood count | 2.00 |
|  | C-reactive protein | 1.80 |
|  | Bronchoalveolar lavage | 0.12 |
|  | Chest X-ray | 1.50 |
|  | Chest tomography | 1.27 |
|  | General ward admission | 6.00 |
|  | Intensive care unit admission | 2.80 |
|  | Mechanical Ventilation | 2.10 |

*Estimated through local expert opinion

# **Market share for the base-case scenario**

## **Table S7.** Market share for the base-case scenario.

| **50-64 years** | **Year 1** | **Year 2** | **Year 3** | **Year 4** | **Year 5** |
| --- | --- | --- | --- | --- | --- |
| **Current scenario** |  |  |  |  |  |
| SD-TIV | 100% | 100% | 100% | 100% | 100% |
| aTIV | 0% | 0% | 0% | 0% | 0% |
| **Projected scenario** |  |  |  |  |  |
| SD-TIV | 94% | 88% | 80% | 70% | 60% |
| aTIV | 6% | 12% | 20% | 30% | 40% |
| **+65 years** | **Year 1** | **Year 2** | **Year 3** | **Year 4** | **Year 5** |
| **Current scenario** |  |  |  |  |  |
| SD-TIV | 45% | 45% | 45% | 45% | 45% |
| aTIV | 55% | 55% | 55% | 55% | 55% |
| **Projected scenario** |  |  |  |  |  |
| SD-TIV | 40% | 35% | 30% | 25% | 20% |
| aTIV | 60% | 65% | 70% | 75% | 80% |

**Source.** Estimated based on expert opinion

# **Market share for the scenario analysis**

Market shares (current and projected) for the different vaccination regimens and age groups (50–64 years with risk factors for influenza and ≥65 years) are discretionary estimates based on information provided by Seqirus and validated by a local expert, reflecting the reality of each health subsector. To estimate market shares for the health system, a weighted average was used based on the coverage rates of each sector (23% social security, 14% private sector, and 63% public sector).

## **Table S8.** Market share for the scenario analysis.

| **50-64 years** | **Year 1** | **Year 2** | **Year 3** | **Year 4** | **Year 5** |
| --- | --- | --- | --- | --- | --- |
| **Current scenario** |  |  |  |  |  |
| SD-TIV | 100% | 100% | 100% | 100% | 100% |
| aTIV | 0% | 0% | 0% | 0% | 0% |
| **Projected scenario** |  |  |  |  |  |
| SD-TIV | 97% | 95% | 90% | 85% | 80% |
| aTIV | 3% | 5% | 10% | 15% | 20% |
| **+65 years** | **Year 1** | **Year 2** | **Year 3** | **Year 4** | **Year 5** |
| **Current scenario** |  |  |  |  |  |
| HD-QIV | 5% | 5% | 5% | 5% | 5% |
| SD-TIV | 95% | 95% | 95% | 95% | 95% |
| aTIV | 0% | 0% | 0% | 0% | 0% |
| **Projected scenario** |  |  |  |  |  |
| HD-QIV | 5% | 5% | 5% | 5% | 5% |
| SD-TIV | 90% | 87% | 83% | 80% | 75% |
| aTIV | 5% | 8% | 12% | 15% | 20% |

**Source.** Estimated based on expert opinion

# **Probabilistic Sensitivity Analysis (PSA)**

## **Table S9. Distribution of variables included in the probabilistic sensitivity analysis**

| **Parameter** | **Distribution** |
| --- | --- |
| Vaccination Coverage Rate 50–64 years high-risk individuals | Beta |
| Vaccination Coverage Rate +65 years high-risk individuals | Beta |
| Age cohort 50-59 years High-risk individuals | Beta |
| Age cohort 60-64 years High-risk individuals | Beta |
| Life expectancy | Gamma |
| Vaccine acquisition cost: aTIV | Gamma |
| Vaccine acquisition cost: TIV-SD | Gamma |
| Medical visit cost | Gamma |
| Hospitalization cost | Gamma |
| Utility general population | Beta |
| Utility lost: Symptomatic case | Beta |
| Duration disutility symptomatic case | Lognormal |
| Utility lost: Medical visit | Beta |
| Duration disutility medical visit | Lognormal |
| Utility lost: Hospitalization | Beta |
| Duration disutility hospitalization | Lognormal |
| SD-TIV efficacy for H1N1 | Lognormal |
| SD-TIV efficacy for H3N2 | Lognormal |
| SD-TIV efficacy for B | Lognormal |
| rVE QIV-HD vs TIV/QIV-SD | Lognormal |
| rVE aTIV vs QIV-HD | Lognormal |
| Probability of hospitalization | Beta |
| Probability of death | Beta |

# **Results for the scenario analysis**

## **Table S10.** **Discounted scenario analysis results.**

| **Influenza outcomes** | **aTIV** | **SD-TIV** | **Difference** |
| --- | --- | --- | --- |
| **Clinical outcomes** |  |  |  |
| Symptomatic cases | 55,201 | 57,350 | -2,149 |
| Medical visits | 26,435 | 27,560 | -1,125 |
| Hospitalizations | 1,374 | 1,414 | -41 |
| Deaths | 173 | 177 | -4 |
| QALYs lost | 1,816.10 | 1,874.10 | -57,93 |
| **Economic outcomes** |  |  |  |
| Vaccine acquisition | $13,350,382 | $12,459,405 | - $890,977 |
| Vaccines administration | $1,498,115 | $1,498,115 | $0 |
| Medical visits | $1,294,785 | $1,241,916 | - $52,869 |
| Hospitalizations | $9,287,479 | $9,019,759 | - $267,721 |

In the alternative scenario for the private healthcare sector, it is estimated that aTIV prevented 2,149 symptomatic influenza cases, 1,125 medical visits, 41 hospitalizations, and 4 deaths across all age groups, compared to the combined strategy SD-TIV for adults 50-64 years and HD-QIV for adults ≥65 years. This strategy would generate savings of $890,977 in vaccination costs, $52,869 in medical visits and $267,721 in hospitalizations.

**Table S10. Alternative scenario results for the costo-effectiveness analysis**

|  | **Total costs** | **QALYs lost** | **Incremental costs** | **Incremental QALYs** | **ICER per QALY** | **Result** |
| --- | --- | --- | --- | --- | --- | --- |
| aTIV | $24,219,194 | -1,816.14 | - $1,211,567 | 57.93 | - | Dominant |
| SD-TIV/ HD-QIV | $25,430,761 | -1,874.07 |  |  |  |  |

## **Figure 1. Tornado diagram for the scenario analysis.**


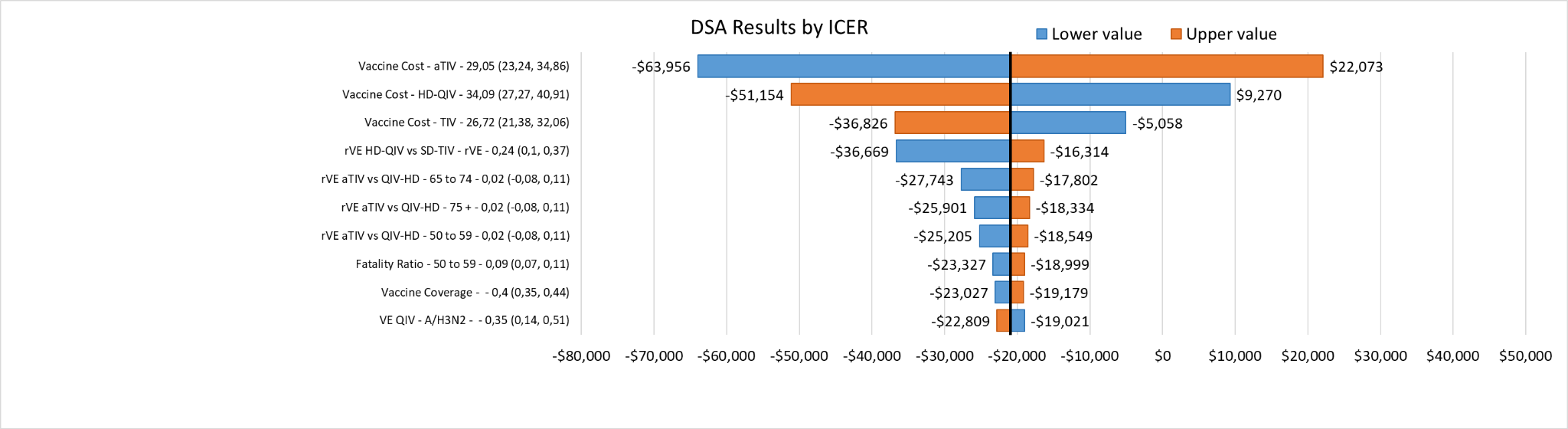


## **Figure 2. Probabilistic sensitivity analysis for the scenario analysis.**


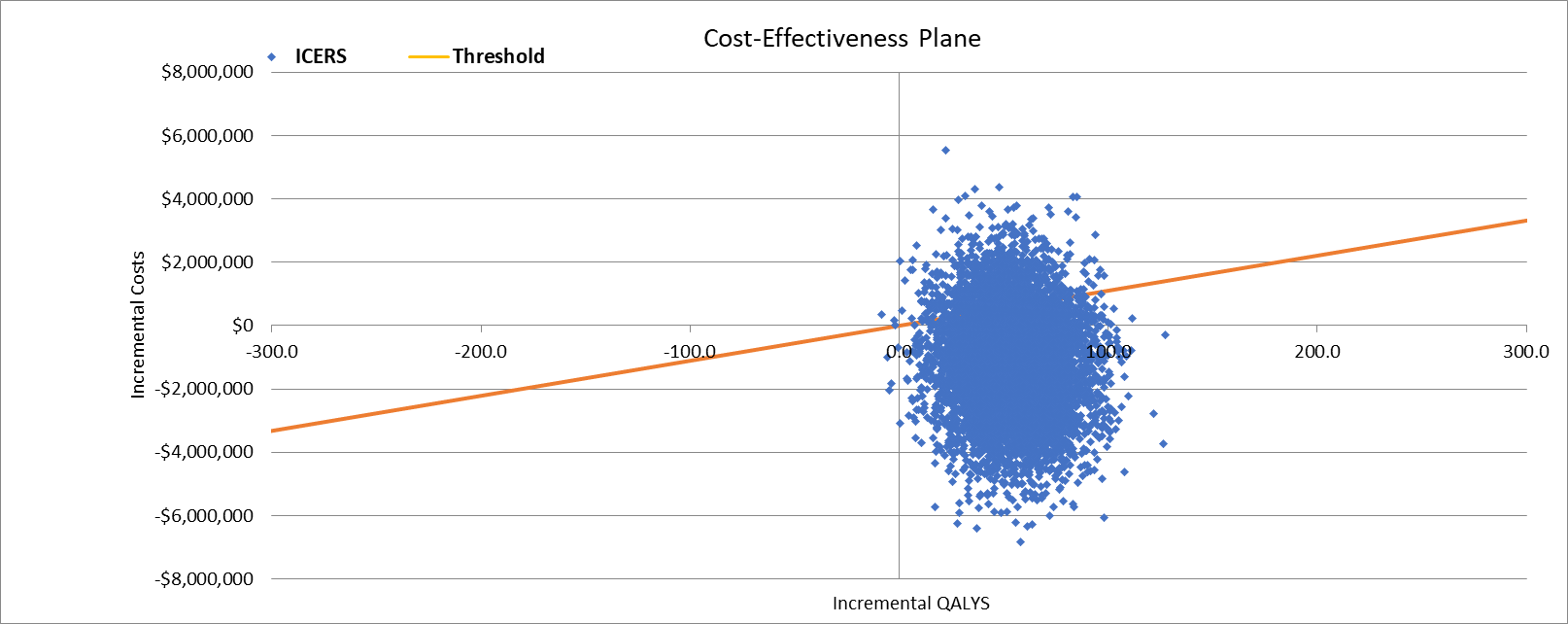


## **Figure 3. Cost-effectiveness acceptability curve for the scenario analysis.**


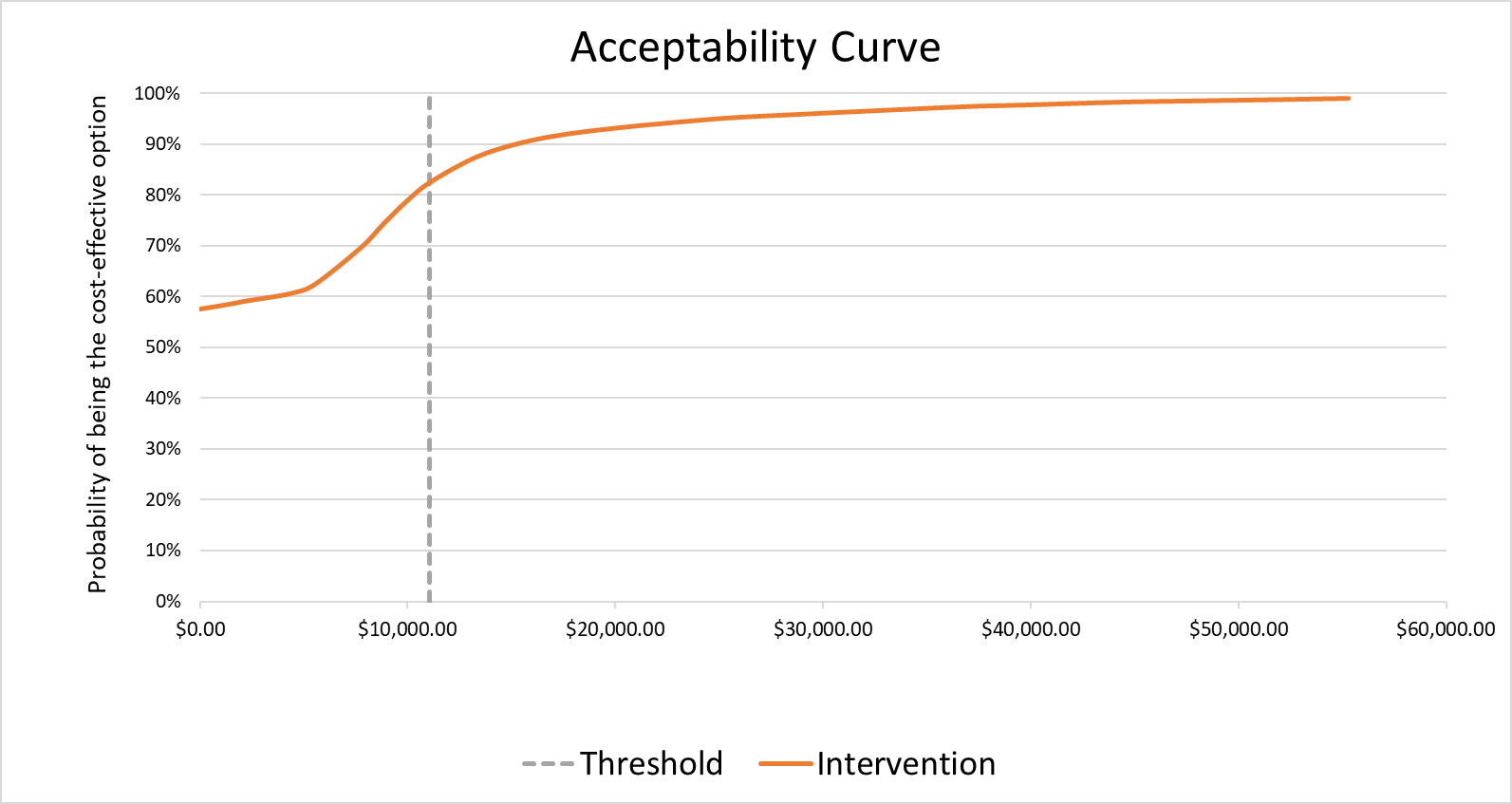


## **Table S11. Budget impact for the scenario analysis.** Costs are reported in March 2025 USD dollars.

|  | **Budget impact** | | | | |  |  |
| --- | --- | --- | --- | --- | --- | --- | --- |
| **Cost component** | Year 1 | Year 2 | Year 3 | Year 4 | Year 5 | Cumulative | Annual average |
| Vaccine acquisition | $42,009 | $68,018 | $112,072 | $150,136 | $200,182 | $572,417 | $114,483 |
| Vaccines administration | $0 | $0 | $0 | $0 | $0 | $0 | $0 |
| Medical visits | - $3,598 | - $5,859 | - $10,064 | - $13,856 | - $18,474 | - $51,852 | - $10,370 |
| Hospitalizations | - $34,470 | - $55,638 | - $89,540 | - $118,009 | - $157,345 | - $455,002 | - $91,000 |
| **Budget impact** | $3,941 | $6,520 | $12,468 | $18,272 | $24,362 | $65,563 | $13,113 |
| **PMPM** | $0.00005 | $0.00008 | $0.00014 | $0.00022 | $0.00028 | $0.00076 | $0.00015 |
| **Budget Impact %** | 0.02% | 0.03% | 0.05% | 0.08% | 0.10% | 0.27% | 0.05% |

**Abbreviations**. *PMPM*, per-member per-months.

## **Figure 4. Budget impact per-member per-month (PMPM) for the scenario analysis.**


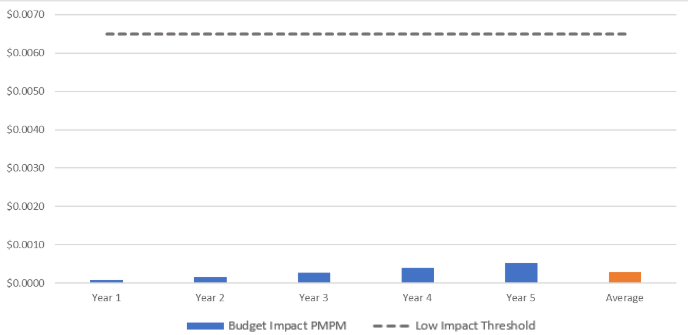


1. [Ma W, Huo X, Zhou M. The healthcare seeking rate of individuals with influenza like illness: a meta-analysis. *Infect Dis (Lond)*. 2018;50(10):728-735. doi:](http://paperpile.com/b/pda1Wq/mzUHE)[10.1080/23744235.2018.1472805](http://dx.doi.org/10.1080/23744235.2018.1472805)

2. [Fleming DM, Taylor RJ, Haguinet F, et al. Influenza-attributable burden in United Kingdom primary care. *Epidemiology & Infection*. 2016;144(3):537-547. doi:](http://paperpile.com/b/pda1Wq/E53cQ)[10.1017/S0950268815001119](http://dx.doi.org/10.1017/S0950268815001119)

3. [Near AM, Tse J, Young-Xu Y, Hong DK, Reyes CM. Burden of influenza hospitalization among high-risk groups in the United States. *BMC Health Serv Res*. 2022;22(1):1209. doi:](http://paperpile.com/b/pda1Wq/rBvXU)[10.1186/s12913-022-08586-y](http://dx.doi.org/10.1186/s12913-022-08586-y)

4. [Mertz D, Kim TH, Johnstone J, et al. Populations at risk for severe or complicated influenza illness: systematic review and meta-analysis. *BMJ*. 2013;347. doi:](http://paperpile.com/b/pda1Wq/3lH8I)[10.1136/bmj.f5061](http://dx.doi.org/10.1136/bmj.f5061)

5. [Belongia EA, Simpson MD, King JP, et al. Variable influenza vaccine effectiveness by subtype: a systematic review and meta-analysis of test-negative design studies. *Lancet Infect Dis*. 2016;16(8):942-951. doi:](http://paperpile.com/b/pda1Wq/ovlBJ)[10.1016/S1473-3099(16)00129-8](http://dx.doi.org/10.1016/S1473-3099(16)00129-8)

6. [Tricco AC, Chit A, Soobiah C, et al. Comparing influenza vaccine efficacy against mismatched and matched strains: a systematic review and meta-analysis. *BMC Med*. 2013;11(1):153. doi:](http://paperpile.com/b/pda1Wq/cAgng)[10.1186/1741-7015-11-153](http://dx.doi.org/10.1186/1741-7015-11-153)

7. [DiazGranados CA, Dunning AJ, Kimmel M, et al. Efficacy of high-dose versus standard-dose influenza vaccine in older adults. *N Engl J Med*. 2014;371(7):635-645. doi:](http://paperpile.com/b/pda1Wq/rwElR)[10.1056/NEJMoa1315727](http://dx.doi.org/10.1056/NEJMoa1315727)

8. [Hsiao A, Yee A, Leong T, et al. 164. Effectiveness of adjuvanted inactivated influenza vaccine versus high-dose inactivated influenza vaccine against PCR-confirmed influenza among adults ≥65 years: A pragmatic randomized study. *Open Forum Infect Dis*. 2025;12(Supplement_1). doi:](http://paperpile.com/b/pda1Wq/cYGxG)[10.1093/ofid/ofae631.001](http://dx.doi.org/10.1093/ofid/ofae631.001)

9. [Bilcke J, Coenen S, Beutels P. Influenza-like-illness and clinically diagnosed flu: disease burden, costs and quality of life for patients seeking ambulatory care or no professional care at all. *PLoS One*. 2014;9(7):e102634. doi:](http://paperpile.com/b/pda1Wq/7pjnt)[10.1371/journal.pone.0102634](http://dx.doi.org/10.1371/journal.pone.0102634)

10. [Hollmann M, Garin O, Galante M, Ferrer M, Dominguez A, Alonso J. Impact of influenza on health-related quality of life among confirmed (H1N1)2009 patients. *PLoS One*. 2013;8(3):e60477. doi:](http://paperpile.com/b/pda1Wq/0Irdn)[10.1371/journal.pone.0060477](http://dx.doi.org/10.1371/journal.pone.0060477)

11. [Grupo AlfaBeta. Precio de medicamentos. Alfa Beta. 2020. Accessed April 2023.](http://paperpile.com/b/pda1Wq/j6RVw) <http://www.alfabeta.net/precio/>

12. [Garfinkel F, Méndez Y. *Value Chain Reports: Health, Pharmacy and Medical Equipment*. Office for Economic Policy and Development Planning; 2016.](http://paperpile.com/b/pda1Wq/ILhgw)

13. [FATSA. *Convenio Salarial*. Federación de Asociaciones de Trabajadores de la Sanidad Argentina; 2025.](http://paperpile.com/b/pda1Wq/H7Klp)
