# Supplementary material for: Cost-Effectiveness and Budget Impact Analysis of the Trivalent Adjuvanted Influenza Vaccine in People over 50 Years of Age for Argentina
Source: Vaccines (Basel). 2026 Feb 28;14(3):227. doi: 10.3390/vaccines14030227 (PMC13030660; doi:10.3390/vaccines14030227)
Supplement: Supplementary file 1 [file vaccines-14-00227-s001.zip › vaccines-4101606-supplementary.pdf]

Supplementary Material **1**
Epidemiological and clinical inputs **1**
Table S1. Probabilities of Medical Visits and Hospitalizations 1
Efficacies inputs **2**
Table S2. Vaccine Efficacies 2
Utilities inputs **2**
Table S3. Utilities lost 2
Cost inputs **3**
Table S4. Cost of drugs included in the management of an outpatient and inpatient event. Costs are reported in March 2025 USD dollars. 3
Table S5. Unit costs of healthcare resources used in the vaccine administration, in the management of an outpatient event and in the management of an inpatient event in Argentina. Costs are reported in March 2025 USD dollars. 3
Table S6. Expected amounts per patient per year of each health and medication used in the vaccines administration, management of an outpatient and inpatient event. Expected quantities are reported according to phase of the health event. 4
Market share for the base-case scenario **5**
Table S7. Market share for the base-case scenario. 5
Market share for the scenario analysis **6**
Table S8. Market share for the scenario analysis. 6
Probabilistic Sensitivity Analysis (PSA) **7**
Table S9. Distribution of variables included in the probabilistic sensitivity analysis 7
Results for the scenario analysis **7**
Table S10. Discounted scenario analysis results. 7
Figure 1. One-way sensitivity analysis for the scenario analysis. 8
Figure 2. Probabilistic sensitivity analysis for the scenario analysis. 9
Figure 3. Cost-effectiveness acceptability curve for the scenario analysis. 9
Table S11. Budget impact for the scenario analysis. Costs are reported in March 2025 USD dollars. 9
Figure 4. Budget impact per-member per-month (PMPM) for the scenario analysis. 10

Supplementary Material

Epidemiological and clinical inputs

Table S1. Probabilities of Medical Visits and Hospitalizations

Parameter	Base Case	Lower bound	Upper bound	Distribution	Reference
**Probability of medical visit***	39%	33%	45%		Ma W, 2018[1] & Fleming, 2016[2]

Parameter	Base Case	Lower bound	Upper bound	Distribution	Reference
**Probability of hospitalization****					
50-64 years	2.3%			Beta distribution	Near, 2022[3] & Mertz, 2013[3,4]
+65 years	9.4%			Beta distribution	

*The probability of medical visit for a symptomatic influenza case was obtained from a meta-analysis conducted in 2018 and was adjusted using the study by Fleming et al. (2016) for the 50–64-year high-risk group for influenza.

**The probability of hospitalization among patients who sought care was obtained from a real-world study conducted in the United States and adjusted for the 50–64-year high-risk group for influenza.

Efficacies inputs

Table S2. Vaccine Efficacies

Parameter	Base Case	Lower bound	Upper bound	Distribution	Reference
**50-64 years**					
SD-TIV efficacy for H1N1	73%	52%	84%	Lognormal distribution	Belongia, 2016[5]
SD-TIV efficacy for H3N2	35%	14%	51%	Lognormal distribution	Belongia, 2016[5]
SD-TIV efficacy for B	77%	18%	94%	Lognormal distribution	Tricco, 2013[6]
QIV-HD vs TIV/QIV-SD for influenza cases	24.2%	9.7%	36.5%	Lognormal distribution	Assumption
aTIV vs QIV-HD for influenza cases	1.5%	-8.4%	10.5%	Lognormal distribution	Assumption
**+65 years**					
SD-TIV efficacy for H1N1	62%	36%	78%	Lognormal distribution	Belongia, 2016[5]
SD-TIV efficacy for H3N2	24%	0%	45%	Lognormal distribution	Belongia, 2016[5]
SD-TIV efficacy for B	53%	39%	64.7%	Lognormal distribution	Tricco, 2013[6]
QIV-HD vs TIV/QIV-SD for influenza cases	24.2%	9.7%	36.5%	Lognormal distribution	DiazGranados, 2014[7]
aTIV vs QIV-HD for influenza cases	1.5%	-8.4%	10.5%	Lognormal distribution	Hsiao, 2025[8]

Utilities inputs

Table S3. Utilities lost

Parameter	Base Case	Lower bound	Upper bound	Distribution	Reference
Symptomatic case	0.30	0.00	0.53	Beta distribution	Bilcke, 2014[9]
**50-64 years**					
Medical visit	0.36	0.29	0.43	Beta distribution	Hollmann, 2013[10]
Hospitalization	0.58	0.49	0.67		Hollmann, 2013[10]

Parameter	Base Case	Lower bound	Upper bound	Distribution	Reference
**+65 years**					
Medical visit	0.32	0.20	0.45	Beta distribution	Hollmann, 2013[10]
Hospitalization	0.56	0.43	0.69		Hollmann, 2013[10]

For the 50–69-year-old group, the utility was estimated as an average of the 45–54 and 55–64 age groups, weighted by the proportion of individuals aged 50–59 who were under 55, based on the 2024 population projections from INDEC.

Cost inputs

Table S4. Cost of drugs included in the management of an outpatient and inpatient event. Costs are reported in March 2025 USD dollars.

Health phases	Drugs	Dosage	Expected amount	Wholesale price per mg*
Management of an outpatient event	Acetaminophen	1 gr every 12 hours	8,400 mg	$0.0002
	Oseltamivir	75 mg per day	26,25 mg	$0.0305
Management of an inpatient event	Piperacillin + tazobactam (IV)	4.5gr every 6 hours	25,200 mg	$0,0121
	Oseltamivir (O)	75 mg per day	197 mg	$0,0305
	Cefepime (IV)	4.5 gr per day	9,000 mg	$0,0248
	Vancomycin (IV)	2 gr per day	5,200 mg	$0,0289
	Sulfamethoxazole + trimethoprim (IV)	1.6 vials of 80/400 mg per day	64 mg	$0,0014
	Ampicillin sulbactam (IV)	1.5 gr every 6 hours	18,000 mg	$0,0076
	Ceftriaxone (IV)	1.5 gr per day	7,350 mg	$0,0117
	Levofloxacin (IV)	500 mg every 12 hours	1,225 mg	$0,0027
	Clarithromycin (IV)	500 mg every 12 hours	2,100 mg	$0,0022

*The retail price was obtained from ALFA BETA, and the ex-factory price was estimated as the retail price divided by 1.7545.[11,12] ** This number is obtained by multiplying the daily dose by the number of days and by the utilization rate.
Abbreviations. IV, Intravenous. O, Orally.

Table S5. Unit costs of healthcare resources used in the vaccine administration, in the management of an outpatient event and in the management of an inpatient event in Argentina. Costs are reported in March 2025 USD dollars.

Health resource	Public/ PAMI sector	Social Security	Private sector	Health System
Consultation with a general practitioner	$9.72	$11.56	$12.42	$10.52

Consultation with pulmonologist	$9.72	$11.56	$12.42	$10.52
Consultation with an infectious disease specialist	$9.72	$11.56	$12.42	$10.52
Vaccine administration*	$0.09	$0.18	$0.23	$0.13
Blood culture	$10.11	$24.83	$26.25	$15.76
Sputum culture	$12.32	$36.52	$38.60	$21.57
Respiratory viral panel	$46.50	$131.48	$138.96	$78.99
Urea	$1.43	$2.19	$2.32	$1.73
Creatinine	$4.07	$2.92	$3.09	$3.67
Hepatogram	$3.71	$8.77	$9.26	$5.65
Blood glucose	$0.78	$2.19	$2.32	$1.32
Ion panel	$2.40	$5.11	$5.40	$3.44
Blood count	$1.62	$4.38	$4.63	$2.68
C-reactive protein	$2.34	$17.53	$18.53	$8.10
Bronchoalveolar lavage	$29.06	$9.27	$15.96	$22.67
Chest X-ray	$6.25	$7.93	$8.79	$6.99
Chest tomography	$49.04	$53.80	$64.60	$52.31
General ward admission	$100.56	$325.40	$358.05	$188.32
Intensive care unit admission	$169.43	$548.27	$618.86	$319.49
Mechanical Ventilation	$185.95	$601.70	$665.66	$348.73

The unit cost of healthcare resources by sector was obtained from the IECS unit cost database.

*The administration cost was calculated based on the wage corresponding to 10 minutes of a nurse's work.[13]

**To estimate the direct medical costs for the health system, a weighted average was used based on the coverage rates of each sector (23% social security, 14% private sector, and 63% public sector).

Table S6. Expected amounts per patient per year of each health and medication used in the vaccines administration, management of an outpatient and inpatient event. Expected quantities are reported according to phase of the health event.

Health phases	Health resource	Expected amounts*
Vaccines administration	Nurse	10.00
Management of an outpatient event	Consultation with a general practitioner	1.50
	Urea	0.40
	Creatinine	0.40
	Hepatogram	0.40
	Blood glucose	0.40

	Ion panel	0.40
	Blood count	0.40
	C-reactive protein	0.40
	Respiratory viral panel	0.01
	Chest X-ray	0.60
	Chest tomography	0.015
Management of an inpatient event	Consultation with pulmonologist	2.00
	Consultation with an infectious disease specialist	4.00
	Consultation with a general practitioner	2.00
	Blood culture	0.90
	Sputum culture	0.40
	Respiratory viral panel	0.35
	Urea	2.00
	Creatinine	2.00
	Hepatogram	2.00
	Blood glucose	2.00
	Ion panel	2.00
	Blood count	2.00
	C-reactive protein	1.80
	Bronchoalveolar lavage	0.12
	Chest X-ray	1.50
	Chest tomography	1.27
	General ward admission	6.00
	Intensive care unit admission	2.80
	Mechanical Ventilation	2.10

*Estimated through local expert opinion

Market share for the base-case scenario

Table S7. Market share for the base-case scenario.

50-64 years	Year 1	Year 2	Year 3	Year 4	Year 5
**Current scenario**					
SD-TIV	100%	100%	100%	100%	100%
aTIV	0%	0%	0%	0%	0%
**Projected scenario**					

50-64 years	Year 1	Year 2	Year 3	Year 4	Year 5
**Current scenario**					
SD-TIV	94%	88%	80%	70%	60%
aTIV	6%	12%	20%	30%	40%
**+65 years**	**Year 1**	**Year 2**	**Year 3**	**Year 4**	**Year 5**
**Current scenario**					
SD-TIV	45%	45%	45%	45%	45%
aTIV	55%	55%	55%	55%	55%
**Projected scenario**					
SD-TIV	40%	35%	30%	25%	20%
aTIV	60%	65%	70%	75%	80%

Source. Estimated based on expert opinion

Market share for the scenario analysis

Market shares (current and projected) for the different vaccination regimens and age groups (50-64 years with risk factors for influenza and ≥65 years) are discretionary estimates based on information provided by Seqirus and validated by a local expert, reflecting the reality of each health subsector. To estimate market shares for the health system, a weighted average was used based on the coverage rates of each sector (23% social security, 14% private sector, and 63% public sector).

Table S8. Market share for the scenario analysis.

50-64 years	Year 1	Year 2	Year 3	Year 4	Year 5
**Current scenario**					
SD-TIV	100%	100%	100%	100%	100%
aTIV	0%	0%	0%	0%	0%
**Projected scenario**					
SD-TIV	97%	95%	90%	85%	80%
aTIV	3%	5%	10%	15%	20%
**+65 years**	**Year 1**	**Year 2**	**Year 3**	**Year 4**	**Year 5**
**Current scenario**					
HD-QIV	5%	5%	5%	5%	5%
SD-TIV	95%	95%	95%	95%	95%
aTIV	0%	0%	0%	0%	0%
**Projected scenario**					
HD-QIV	5%	5%	5%	5%	5%

50-64 years	Year 1	Year 2	Year 3	Year 4	Year 5
**Current scenario**					
SD-TIV	90%	87%	83%	80%	75%
aTIV	5%	8%	12%	15%	20%

Source. Estimated based on expert opinion

Probabilistic Sensitivity Analysis (PSA)

Table S9. Distribution of variables included in the probabilistic sensitivity analysis

Parameter	Distribution
Vaccination Coverage Rate 50–64 years high-risk individuals	Beta
Vaccination Coverage Rate +65 years high-risk individuals	Beta
Age cohort 50-59 years High-risk individuals	Beta
Age cohort 60-64 years High-risk individuals	Beta
Life expectancy	Gamma
Vaccine acquisition cost: aTIV	Gamma
Vaccine acquisition cost: TIV-SD	Gamma
Medical visit cost	Gamma
Hospitalization cost	Gamma
Utility general population	Beta
Utility lost: Symptomatic case	Beta
Duration disutility symptomatic case	Lognormal
Utility lost: Medical visit	Beta
Duration disutility medical visit	Lognormal
Utility lost: Hospitalization	Beta
Duration disutility hospitalization	Lognormal
SD-TIV efficacy for H1N1	Lognormal
SD-TIV efficacy for H3N2	Lognormal
SD-TIV efficacy for B	Lognormal
rVE QIV-HD vs TIV/QIV-SD	Lognormal
rVE aTIV vs QIV-HD	Lognormal
Probability of hospitalization	Beta
Probability of death	Beta

Results for the scenario analysis

Table S10. Discounted scenario analysis results.

Influenza outcomes	aTIV	SD-TIV	Difference
**Clinical outcomes**			
Symptomatic cases	55,201	57,350	-2,149
Medical visits	26,435	27,560	-1,125
Hospitalizations	1,374	1,414	-41
Deaths	173	177	-4
QALYs lost	1,816.10	1,874.10	-57,93
**Economic outcomes**			
Vaccine acquisition	$13,350,382	$12,459,405	- $890,977
Vaccines administration	$1,498,115	$1,498,115	$0
Medical visits	$1,294,785	$1,241,916	- $52,869
Hospitalizations	$9,287,479	$9,019,759	- $267,721

In the alternative scenario for the private healthcare sector, it is estimated that aTIV prevented 2,149 symptomatic influenza cases, 1,125 medical visits, 41 hospitalizations, and 4 deaths across all age groups, compared to the combined strategy SD-TIV for adults 50-64 years and HD-QIV for adults ≥65 years. This strategy would generate savings of $890,977 in vaccination costs, $52,869 in medical visits and $267,721 in hospitalizations.

Table S10. Alternative scenario results for the costo-effectiveness analysis

	Total costs	QALYs lost	Incremental costs	Incremental QALYs	ICER per QALY	Result
aTIV	$24,219,194	-1,816.14	- $1,211,567	57.93	-	Dominant
SD-TIV/ HD-QIV	$25,430,761	-1,874.07				

Figure 1. Tornado diagram for the scenario analysis.

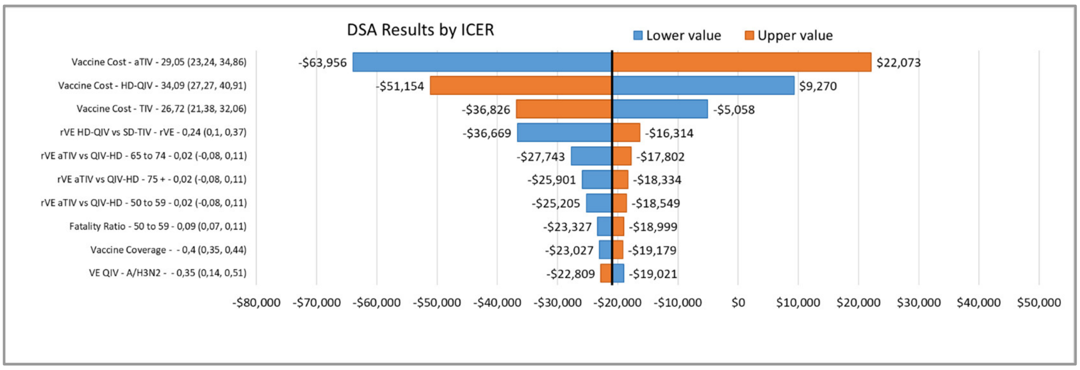

Figure 2. Probabilistic sensitivity analysis for the scenario analysis.

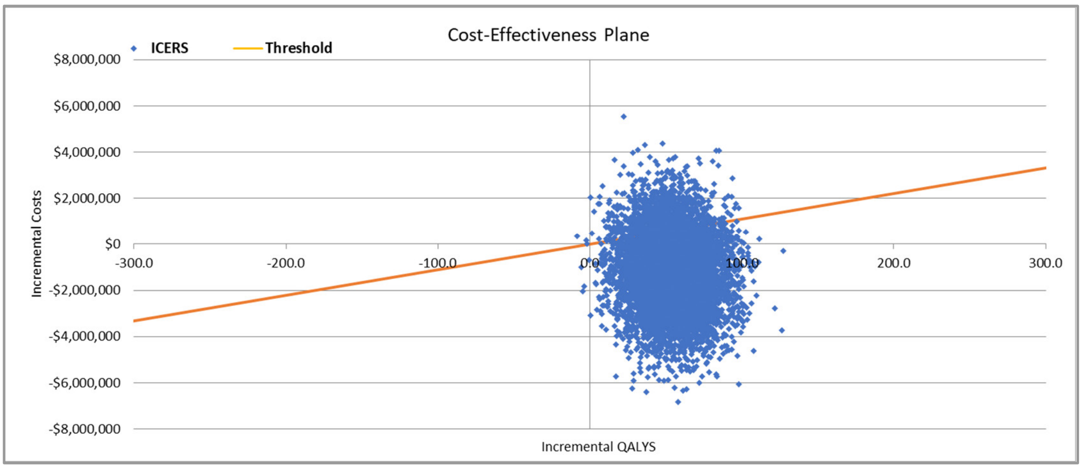

Figure 3. Cost-effectiveness acceptability curve for the scenario analysis.

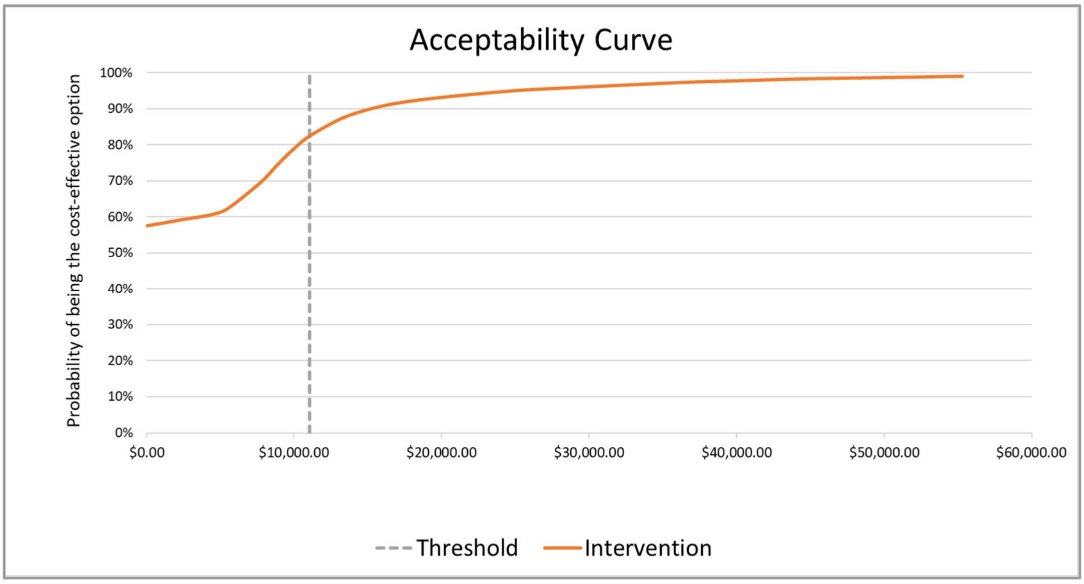

Table S11. Budget impact for the scenario analysis. Costs are reported in March 2025 USD dollars.

	Budget impact						
**Cost component**	Year 1	Year 2	Year 3	Year 4	Year 5	Cumulative	Annual average
Vaccine acquisition	$42,009	$68,018	$112,072	$150,136	$200,182	$572,417	$114,483
Vaccines administration	$0	$0	$0	$0	$0	$0	$0
Medical visits	- $3,598	- $5,859	- $10,064	- $13,856	- $18,474	- $51,852	- $10,370
Hospitalizations	- $34,470	- $55,638	- $89,540	- $118,009	- $157,345	- $455,002	- $91,000
**Budget impact**	$3,941	$6,520	$12,468	$18,272	$24,362	$65,563	$13,113
**PMPM**	$0.00005	$0.00008	$0.00014	$0.00022	$0.00028	$0.00076	$0.00015
**Budget Impact %**	0.02%	0.03%	0.05%	0.08%	0.10%	0.27%	0.05%

Abbreviations. *PMPM*, per-member per-months.

Figure 4. Budget impact per-member per-month (PMPM) for the scenario analysis.

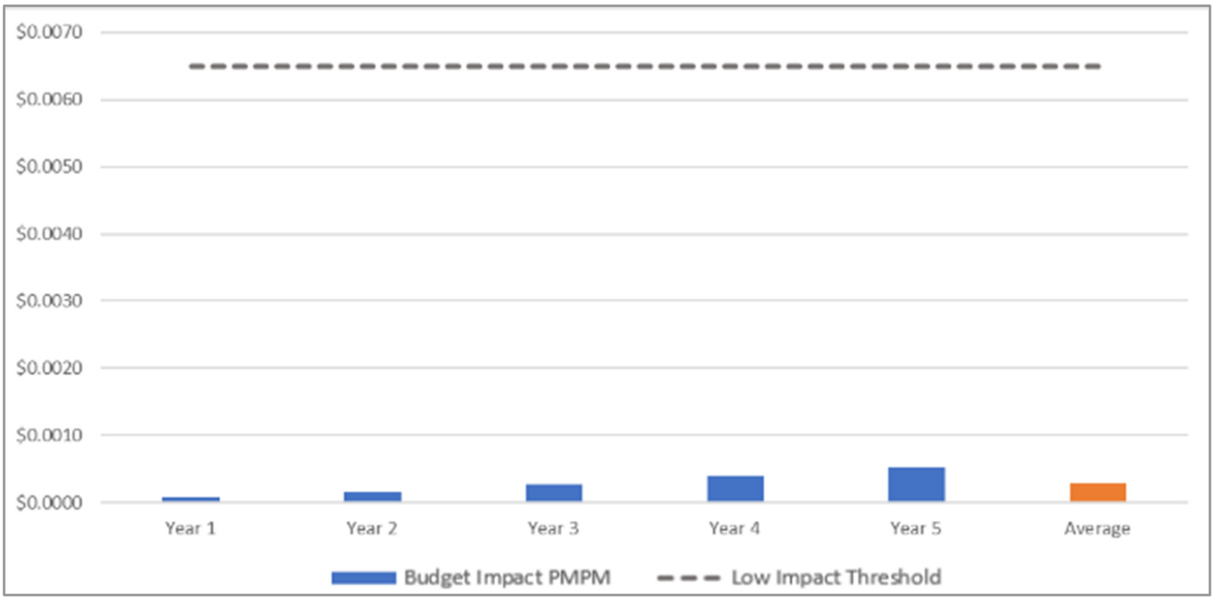

1. Ma W, Huo X, Zhou M. The healthcare seeking rate of individuals with influenza like illness: a meta-analysis. *Infect Dis (Lond)*. 2018;50(10):728-735. doi:10.1080/23744235.2018.1472805

2. Fleming DM, Taylor RJ, Haguinet F, et al. Influenza-attributable burden in United Kingdom primary care. *Epidemiology & Infection*. 2016;144(3):537-547. doi:10.1017/S0950268815001119

3. Near AM, Tse J, Young-Xu Y, Hong DK, Reyes CM. Burden of influenza hospitalization among high-risk groups in the United States. *BMC Health Serv Res*. 2022;22(1):1209. doi:10.1186/s12913-022-08586-y

4. Mertz D, Kim TH, Johnstone J, et al. Populations at risk for severe or complicated influenza illness: systematic review and meta-analysis. *BMJ*. 2013;347. doi:10.1136/bmj.f5061

5. Belongia EA, Simpson MD, King JP, et al. Variable influenza vaccine effectiveness by subtype: a systematic review and meta-analysis of test-negative design studies. *Lancet Infect Dis*. 2016;16(8):942-951. doi:10.1016/S1473-3099(16)00129-8

6. Tricco AC, Chit A, Soobiah C, et al. Comparing influenza vaccine efficacy against mismatched and matched strains: a systematic review and meta-analysis. *BMC Med*. 2013;11(1):153. doi:10.1186/1741-7015-11-153

7. DiazGranados CA, Dunning AJ, Kimmel M, et al. Efficacy of high-dose versus standard-dose influenza vaccine in older adults. *N Engl J Med*. 2014;371(7):635-645. doi:10.1056/NEJMoa1315727

8. Hsiao A, Yee A, Leong T, et al. 164. Effectiveness of adjuvanted inactivated influenza vaccine versus high-dose inactivated influenza vaccine against PCR-confirmed influenza among adults ≥65 years: A pragmatic randomized study. *Open Forum Infect Dis*. 2025;12(Supplement_1). doi:10.1093/ofid/ofae631.001

9. Bilcke J, Coenen S, Beutels P. Influenza-like-illness and clinically diagnosed flu: disease burden, costs and quality of life for patients seeking ambulatory care or no professional care at all. *PLoS One*. 2014;9(7):e102634. doi:10.1371/journal.pone.0102634

10. Hollmann M, Garin O, Galante M, Ferrer M, Dominguez A, Alonso J. Impact of influenza on health-related quality of life among confirmed (H1N1)2009 patients. *PLoS One*. 2013;8(3):e60477. doi:10.1371/journal.pone.0060477

11. Grupo AlfaBeta. Precio de medicamentos. Alfa Beta. 2020. Accessed April 2023. http://www.alfabeta.net/precio/

12. Garfinkel F, Méndez Y. *Value Chain Reports: Health, Pharmacy and Medical Equipment*. Office for Economic Policy and Development Planning; 2016.

13. FATSA. *Convenio Salarial*. Federación de Asociaciones de Trabajadores de la Sanidad Argentina; 2025.
